# Systematic Evaluation of Mesophyll Protoplast Isolation and PEG-Mediated Transient Expression in *Rosa chinensis* ‘Old Blush’

**DOI:** 10.3390/plants15182739

**Published:** 2026-09-08

**Authors:** Yiyang Zhao, Yin Zhong, Fangmei Hou, Yousry A. El-Kassaby, Deqiang Zhang

**Affiliations:** 1 College of Landscape Architecture, Beijing University of Agriculture, Beijing 102206, China; yvonne0929@126.com (Y.Z.);; 2 Department of Forest and Conservation Sciences, Faculty of Forestry, Forest Sciences Centre, University of British Columbia, Vancouver, BC V6T 1Z4, Canada

**Keywords:** *Rosa chinensis*, mesophyll protoplast, protoplast isolation, PEG-mediated transfection, transient expression, Cellulase R-10, mGFP6

## Abstract

Stable transformation in rose is slow and genotype-dependent, motivating rapid cellular assays for candidate-gene testing. We systematically evaluated mesophyll protoplast isolation and PEG-mediated transient expression in *Rosa chinensis* ‘Old Blush’. Sequential experiments compared enzyme preparations, digestion time, D-mannitol concentration, cultivar, and donor cultivation regime. Under the tested conditions, Yakult preparations containing 3.0% Cellulase R-10 and 2.0% Macerozyme R-10, 16 h digestion, and 0.4 mol/L D-mannitol yielded 4.641 ± 0.102 × 10^6^ protoplasts/g fresh weight with 94.26 ± 0.15% FDA viability. Recovery varied significantly among donor regimes and between the two cultivars tested. In a 27-combination factorial experiment with three plants as blocks, 25% PEG 4000 working solution (12.5% nominal final concentration), 8 min transfection, and 20 h dark incubation gave the highest observed mGFP6-positive fraction (7.14 ± 0.22%). This efficiency supports imaging of individually transfected cells under the tested conditions, but broader population-level or high-throughput applications require further validation. Protoplast division, plant regeneration, stable transformation, cross-date reproducibility, and transfer to other genotypes were not assessed.

## 1. Introduction

Rose is a major ornamental crop and a perennial woody system for studying recurrent flowering, floral organ development, fragrance and pigment metabolism, stress responses, and domestication-related traits. The chromosome-scale genome of *Rosa chinensis* ‘Old Blush’ supports candidate-gene and regulatory-locus discovery [1], but functional validation remains slower because stable transformation depends on genotype-responsive regeneration, prolonged tissue culture, and recovery of transformed plantlets [2].

Stable transformation is required for heritable modification and whole-plant phenotyping, but it is inefficient as an initial screen for every candidate gene. Somatic embryogenesis and Agrobacterium-mediated transformation have been demonstrated in diploid ‘Old Blush’, although these procedures are labor-intensive and do not transfer equally among rose genotypes [2]. Protoplast assays can provide faster preliminary evidence for reporter expression and imaging. However, an efficiency sufficient to identify individually fluorescent cells should not be assumed to support population-level biochemical measurements, high-throughput promoter assays, or genome-editing evaluation without application-specific validation.

Removing the cell wall allows plant protoplasts to take up nucleic acids and other macromolecules [3]. Mesophyll protoplasts are widely used for rapid transient-expression and signal-response assays in herbaceous models [4] and increasingly support synthetic-biology and genome-editing workflows [5]. Their short assay time is useful for woody ornamentals, in which generation time and tissue-culture recalcitrance slow stable transformation [6].

Protoplast protocols do not transfer directly across species. Release and viability depend on tissue age, donor physiology, cell-wall properties, enzyme activity, osmotic balance, and physical handling. Earlier Rosa studies established mesophyll protoplast isolation and culture [7], examined genotype-dependent isolation and regeneration [8], or regenerated plants from embryogenic-cell-derived protoplasts [9]. These systems were tissue- and genotype-dependent, often required established embryogenic cultures or lengthy regeneration, and did not systematically compare enzyme preparation sources, donor cultivation regimes, and a full-factorial PEG transient-expression design in mesophyll protoplasts. These limitations motivated an explicitly bounded evaluation in ‘Old Blush’.

Isolation conditions must balance yield and viability. High recovery can coincide with membrane damage, debris, or rapid viability loss, whereas gentle digestion may release too few cells for transfection. FDA staining provides a practical viability readout [10], but it should be interpreted with yield, morphology, and downstream expression. Osmotic conditions also require control because hypo-osmotic or hyperosmotic media can cause rupture, shrinkage, or irregular morphology [11].

PEG-mediated DNA delivery depends on protoplast density, plasmid amount and quality, PEG concentration, calcium-containing buffer composition, exposure time, and post-transfection incubation [4,12,13,14,15,16]. Intermediate PEG and incubation conditions often outperform stronger treatments [12,13,14,15,16], consistent with the need to permit DNA uptake while retaining enough viable cells for reporter detection. An integrated isolation and transfection operating range has not been established for rose mesophyll protoplasts.

We evaluated a mesophyll protoplast isolation and transient-expression workflow for *Rosa chinensis* ‘Old Blush’. Earlier Rosa studies established isolation, culture, or regeneration from mesophyll and embryogenic tissues [7,8,9], but those systems were strongly dependent on tissue source and genotype and did not address the combined enzyme-source, donor-regime, and full-factorial PEG questions examined here. Woody-plant studies likewise show that donor tissue, enzyme formulation, digestion time, and osmotic conditions require species-specific optimization [17,18,19]. Our results therefore identify selected operating conditions for the tested material rather than a universal rose protocol.

## 2. Materials and Methods

### 2.1. Plant Materials and Growth Conditions

*Rosa chinensis* ‘Old Blush’ was the principal experimental genotype. Three donor cultivation regimes were evaluated: outdoor field-grown plants, in vitro plantlets, and greenhouse-grown cuttings. Field material was collected from three-year-old cutting-propagated plants. Young, fully expanded leaves were sampled from the second or third node below the shoot apex. Leaves were approximately 15 d old, free of visible disease or mechanical damage, and had not reached the leathery stage.

In vitro plantlets were established from surface-sterilized nodal explants and maintained on half-strength Murashige and Skoog medium containing 3.0 mg/L 6-benzylaminopurine, 0.05 mg/L 1-naphthaleneacetic acid, 3% (*w*/*v*) sucrose, and 0.62% (*w*/*v*) agar at pH 5.8. Cultures were maintained at 25 ± 2 °C under a 16 h light/8 h dark photoperiod and a light intensity of 2000–3000 lx. Plantlets were subcultured every 25–30 d and sampled after approximately 60 d.

Greenhouse cuttings were prepared from healthy one-year-old shoots with two nodes, transferred after rooting to a 2:3:1 (*v*/*v*/*v*) vermiculite:peat:perlite mixture, and grown at 20–25 °C for 60–90 d before sampling. The cultivar ‘Blue Rhapsody’ was propagated only under the same greenhouse conditions and was used exclusively for the genotype comparison, in which all nine enzyme concentration combinations were evaluated. It was not included in the field-grown versus in vitro versus greenhouse donor-regime comparison; that experiment used ‘Old Blush’ alone. Donor materials were sampled in the same season and experimental period.

### 2.2. Experimental Design

The isolation workflow was evaluated in sequential experiments. Cellulase R-10 was tested at 2.0%, 3.0%, and 4.0% (*w*/*v*), and Macerozyme R-10 at 1.0%, 1.5%, and 2.0% (*w*/*v*). All nine concentration combinations were evaluated separately using enzyme preparations obtained from Yakult Pharmaceutical Industry Co., Ltd. (Cellulase “Onozuka” R-10, lot 250124-01; Macerozyme R-10, lot 230801-02, Tokyo, Japan) and Coolaber Science & Technology (Beijing, China). Subsequent experiments used the Yakult preparations unless otherwise stated.

Digestion time was evaluated at 6, 8, 10, 12, 14, 16, 18, 20, 22, and 24 h. D-mannitol was tested at 0.3, 0.4, 0.5, 0.6, 0.7, and 0.8 mol L^−1^. The genotype comparison was conducted by applying all nine enzyme concentration combinations to greenhouse-grown ‘Old Blush’ and ‘Blue Rhapsody’. The donor cultivation-regime comparison was conducted by applying the same matrix to field-grown, in vitro, and greenhouse-grown ‘Old Blush’.

PEG-mediated transfection was evaluated using a three-factor, three-level full-factorial design. PEG 4000 working-solution concentration was tested at 20%, 25%, and 30% (*w*/*v*), corresponding to nominal final concentrations of 10%, 12.5%, and 15%, respectively, after equal-volume mixing with the protoplast/DNA mixture. Transfection time was 4, 8, or 12 min, and dark post-transfection incubation was 15, 20, or 25 h. All 27 combinations were applied to aliquots derived from each of the same three individual plants sampled on the same date. Each plant × treatment combination constituted one of three independent biological replicates, and plant served as a block. The evaluated factors and levels are summarized in Table 1.

### 2.3. Enzyme Solutions and Buffers

The standard enzyme solution contained 20 mmol/L MES, pH 5.7, 20 mmol/L KCl, 10 mmol/L CaCl_2_·2H_2_O, 0.1% (*w*/*v*) bovine serum albumin, 0.01% (*w*/*v*) polyvinylpyrrolidone, D-mannitol at the concentration specified for each experiment, and the designated concentrations of Cellulase R-10 and Macerozyme R-10. For the standard workflow, D-mannitol was used at 0.4 mol/L. Solutions containing the enzymes, mannitol, KCl, and MES were incubated at 55 °C for 10 min, cooled to room temperature, and supplemented with CaCl_2_·2H_2_O, bovine serum albumin, and polyvinylpyrrolidone immediately before use.

W5 solution contained 154 mmol/L NaCl, 125 mmol/L CaCl_2_, 5 mmol/L KCl, and 2 mmol/L MES at pH 5.7. MMG buffer contained 0.4 mol/L D-mannitol, 15 mmol/L MgCl_2_, and 4 mmol/L MES, was adjusted to pH 5.7, and was sterile-filtered before use.

A sterile-filtered 60% PEG 4000 stock solution (Coolaber Science & Technology, Beijing, China, SL9512-100mL, lot SL35171701) was diluted with sterile MMG buffer to prepare the PEG 4000 working solutions. For 3.00 mL of 20%, 25%, or 30% (*w*/*v*) working solution, 1.00, 1.25, or 1.50 mL of 60% PEG 4000 stock was mixed with 2.00, 1.75, or 1.50 mL of MMG buffer, respectively. These percentages refer to the working solutions before addition to the protoplast/DNA mixture.

### 2.4. Mesophyll Protoplast Isolation and Purification

**Figure 1:**
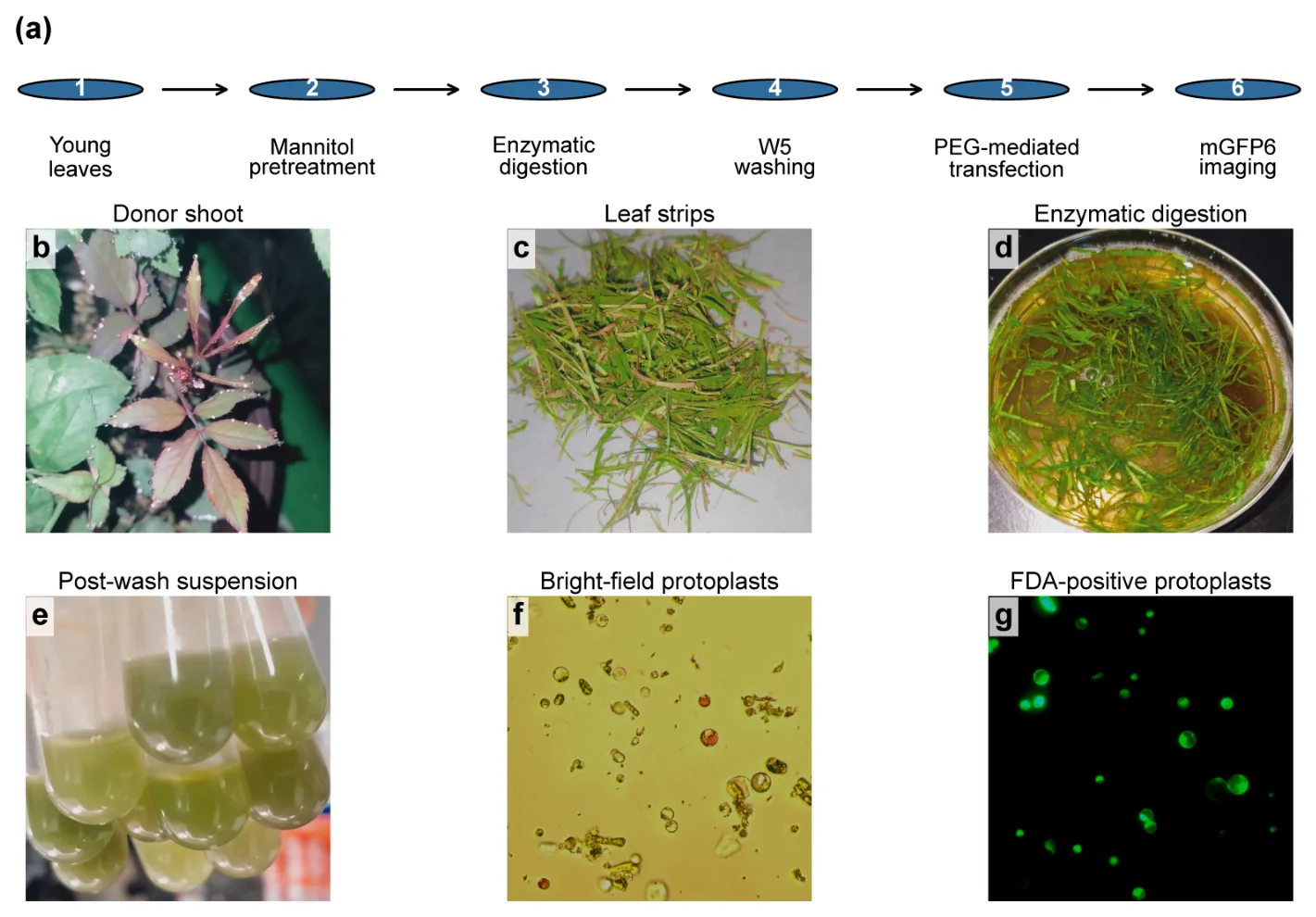
Overview of the evaluated mesophyll protoplast workflow. (**a**) Experimental workflow from donor-leaf selection to mGFP6 readout. (**b**–**g**) Representative images showing donor shoot selection, leaf-strip preparation, enzymatic digestion, post-wash protoplast suspension, bright-field protoplast morphology, and FDA-positive viable protoplasts. Scale bars in (**f**,**g**), 100 μm.

Leaf margins and midveins were removed with a sterile razor blade. The remaining lamina was cut into approximately 1 mm strips and incubated in 0.4 mol/L D-mannitol for 30 min. Pretreated tissue was transferred to enzyme solution at 1 g fresh tissue per 10 mL solution. Samples were vacuum infiltrated at 0.05 MPa for 1 h in darkness at 25–28 °C and then incubated in darkness on a rotary shaker at 40–50 rpm for the specified digestion time. The workflow is summarized in Figure 1.

Digestion was terminated by adding an equal volume of W5 solution. The suspension was gently mixed, incubated in darkness for 10 min, and filtered through a 70 μm cell strainer into a pre-chilled 50 mL tube. Protoplasts were collected by centrifugation at 100× *g* for 5 min at 4 °C. The supernatant was removed, and the pellet was gently resuspended and washed twice with pre-chilled W5 solution. Purified protoplasts were finally resuspended in 5 mL W5 and kept on ice for 30 min before counting or downstream use.

### 2.5. Protoplast Yield and Viability

Protoplast yield was measured with a hemocytometer. A 10 μL aliquot of a well-mixed suspension was loaded into the counting chamber, and protoplasts in the central and four corner squares were counted. Yield was expressed as protoplasts per gram of leaf fresh weight.

Viability was assessed using fluorescein diacetate (FDA) staining [10]. A 2% (*w*/*v*) FDA stock was prepared in acetone and diluted 1:100 in 0.8 mol/L D-mannitol immediately before use. Equal volumes of protoplast suspension and FDA working solution were mixed and incubated for 5–10 min at room temperature in darkness. Images were acquired within 30 min. Protoplasts showing yellow-green fluorescence were scored as viable, and viability was calculated as the number of fluorescent protoplasts divided by the total number observed in the corresponding bright-field image.

Bright-field morphology and FDA fluorescence were imaged using a WY-900 research-grade inverted biological microscope (VIYEE Optoelectronics (Tianjin) Co., Ltd., Tianjin, China) equipped with an E3ISPM20000KPA camera (ToupTek, Hangzhou, China). Image preview and capture were performed at 5440 × 3648 pixels in RGB24 format with automatic exposure disabled. The exposure target was 120 and camera gain was set to 100% throughout. Bright-field images were acquired with a 10 ms exposure, white-balance temperature of 4209, and tint of 1149. FDA fluorescence was excited with a 488 nm light source, and emission was collected at 500–540 nm using a 4880 ms exposure, white-balance temperature of 4209, tint of 1026, hue of 1, saturation of 127, brightness of −41, contrast of 91, and gamma of 1.58.

### 2.6. Plasmid Preparation

A laboratory-provided pMDC85-1 derivative (12,462 bp) carrying mGFP6 was obtained from the authors’ laboratory collection and propagated in Escherichia coli. The T-DNA expression region contains a CaMV 35S promoter, a Gateway recombination cassette, and an mGFP6–6 × His reporter followed by the nos terminator; mGFP6 was used as a stand-alone reporter and was not fused to a rose protein. The vector carries hygromycin resistance for plant selection, kanamycin resistance for bacterial selection, and the chloramphenicol-resistance/ccdB Gateway cassette [20]. A sequence-derived vector map is provided as Supplementary Figure S2, and the complete annotated plasmid sequence is provided as Supplementary Data S1. Plasmid DNA was purified using a commercial plasmid miniprep kit (Tiangen Biotech, Beijing, China), had an A260/A280 ratio of 1.8–2.0, and was adjusted to 1 µg/µL; 10 µg was used per transfection. The matched negative-control plasmid was the corresponding laboratory-stock pMDC85-1 backbone without the mGFP6 coding sequence. Its historical cloning steps were not part of this study.

### 2.7. PEG-Mediated Transfection and Post-Transfection Incubation

Purified protoplasts with an initial FDA viability of at least 80% were resuspended in MMG buffer at 2 × 10^5^ protoplasts/mL. A 100 µL aliquot, containing approximately 2 × 10^4^ protoplasts, was transferred to a 2 mL tube and gently mixed with 10 µL of plasmid DNA. After 3 min on ice, 110 µL of 20%, 25%, or 30% PEG 4000 working solution was added. Because the PEG working solution was mixed with an equal volume (110 µL) of protoplast/DNA mixture, the corresponding nominal final PEG concentrations in the 220 µL reaction were 10%, 12.5%, and 15%, respectively. Tubes were gently inverted one to three times and incubated in darkness for 4, 8, or 12 min.

Transfection was terminated by adding 440 μL pre-chilled W5 solution. Samples were gently mixed and centrifuged at 100× *g* for 5 min at 4 °C. The supernatant was removed, and protoplasts were gently resuspended in 1 mL W5 solution. Samples were incubated horizontally at approximately 23 °C in darkness for 15, 20, or 25 h without agitation.

### 2.8. Fluorescence Imaging and Transient Expression Efficiency

After incubation, protoplasts were collected by centrifugation at 100× *g* for 5 min at 4 °C and gently resuspended. A 10 µL aliquot was mounted in a chamber that prevented compression by the coverslip. Living protoplasts were examined using a ZEISS LSM 910 confocal laser-scanning microscope (Carl Zeiss Microscopy GmbH, Jena, Germany) equipped with an Axio Observer 7 (Carl Zeiss Microscopy GmbH, Jena, Germany) inverted microscope stand and a 40×/1.2 NA water-immersion objective (Carl Zeiss Microscopy GmbH, Jena, Germany). Images were acquired using ZEN 3.12 (Carl Zeiss Microscopy GmbH, Jena, Germany) with the pinhole set to 1 Airy unit.

The 488 nm laser was used to excite mGFP6 fluorescence, which was detected at 490–570 nm; the 561 nm laser was used to excite chloroplast red autofluorescence, detected at 565–700 nm. The fluorescence channels were scanned separately using the Best Signal setting. Images were acquired at 512 × 512 pixels with a scan speed of 700 Hz, line averaging of 2, a master gain of 700 V, and fixed laser-power settings within the confirmed 0.5–3% range. During batch acquisition of all samples, the 488 nm and 561 nm laser power, detector gain, offset, pinhole size, and scan parameters were held constant; no laser intensity or acquisition parameter was adjusted for individual samples. Transmitted-light images were collected using the ESID setting at 400 nm.

Three fields were evaluated for each plant × treatment biological replicate and summarized to one plant × treatment value, giving nine fields per treatment across the three independent biological replicates. Transient expression efficiency was the percentage of protoplasts in those fields scored as mGFP6 positive. A protoplast was scored as positive when visible green fluorescence was present in the GFP channel relative to both untransformed protoplasts and matched pMDC85-1 empty-vector-transfected protoplasts used as negative controls. Post-transfection viable-cell abundance was assessed by FDA staining with an inverted fluorescence microscope at 20× magnification and reported as FDA-positive protoplasts per imaged field. The recorded FDA endpoint did not include a matched total-cell denominator; therefore, a post-transfection FDA-positive percentage or survival percentage could not be calculated retrospectively.

### 2.9. Statistical Analysis

Data are presented as mean ± standard deviation from three plant-level biological replicates. For isolation experiments, technical counts within each plant were averaged before statistical analysis, so plant was the biological unit (n = 3 per condition). Enzyme-source, cultivar, and donor-regime experiments were analyzed by fixed-effect factorial ANOVA including Cellulase R-10, Macerozyme R-10, the grouping factor, and all interactions. Digestion-time and D-mannitol experiments were analyzed by one-way ANOVA followed by Duncan’s multiple range test. Normality and homogeneity of variance were evaluated using the Shapiro–Wilk and Levene tests, respectively. Differences were considered significant at *p* < 0.05; complete isolation ANOVA results are provided in Supplementary Table S7. For the transfection analysis, percentages were converted to proportions and arcsine-square-root transformed; untransformed percentages are shown for interpretability. FDA-positive counts per field are reported on the original count scale.

Statistical analyses were conducted using SPSS Statistics for Windows, version 18.0 (SPSS Inc., Chicago, IL, USA). Transient expression efficiency was analyzed by balanced three-way factorial ANOVA with PEG 4000 working-solution concentration, transfection time, and dark incubation time, including all two-way and three-way interactions, as fixed effects; plant was included as a fixed block. The three field observations for each plant × treatment combination were summarized to one value before analysis. Pearson and Spearman correlations between FDA-positive protoplast counts per field and transient expression efficiency were calculated descriptively across the 27 treatment-level means. Final figure preparation used Python 3.12 and Matplotlib 3.11.1.

## 3. Results

### 3.1. Effects of Enzyme Preparation Source and Concentration on Protoplast Recovery

The two tested enzyme preparations produced significantly different isolation outcomes across the same Cellulase R-10 and Macerozyme R-10 concentration matrix. Factorial ANOVA detected a source effect for both yield (*F*1,36 = 19,692.04, *p* < 0.001) and viability (*F*1,36 = 19,827.72, *p* < 0.001), together with significant source × enzyme interactions (Supplementary Table S7a). With Yakult preparations, yields ranged from 2.164 × 10^6^ to 4.641 × 10^6^ protoplasts/g fresh weight, and viability remained between 88.72% and 97.15%. The highest yield, 4.641 ± 0.102 × 10^6^ protoplasts/g fresh weight, was obtained with 3.0% Cellulase R-10 and 2.0% Macerozyme R-10; viability under this condition was 94.26 ± 0.15%. Complete condition-level values are provided in Supplementary Table S1. The selected operating conditions and outcomes across the complete workflow are summarized in Table 2.

**Figure 2:**
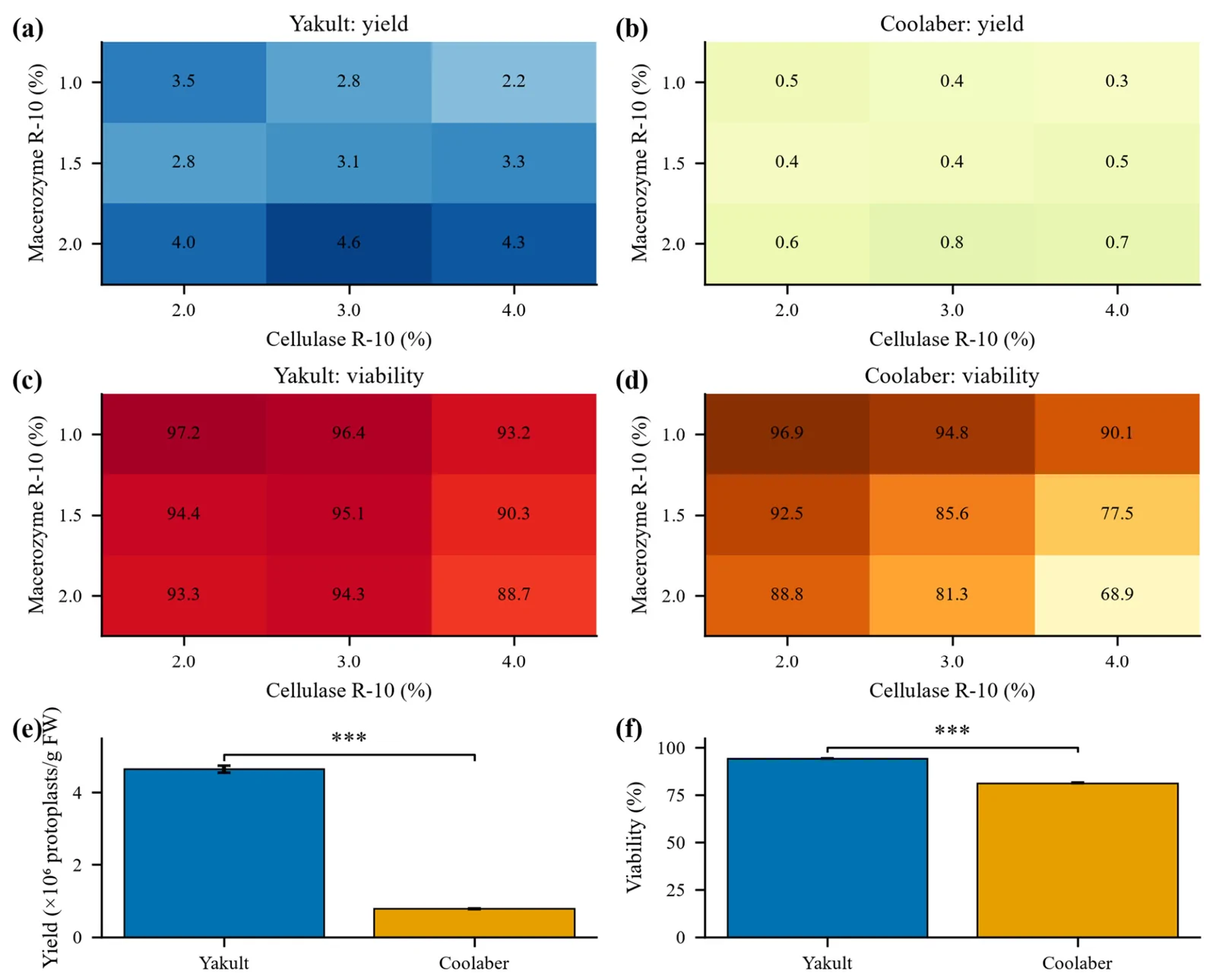
Protoplast recovery differed between the tested enzyme preparations. (**a**,**b**) Protoplast yield matrices obtained with Yakult and Coolaber enzyme preparations, respectively. (**c**,**d**) FDA viability matrices obtained with Yakult and Coolaber preparations, respectively. (**e**) Maximum yield comparison between preparation sources. (**f**) FDA viability comparison under the selected 3.0% Cellulase R-10 and 2.0% Macerozyme R-10 formulation. Data are mean ± SD from three plant-level biological replicates. *** *p* < 0.001 for the displayed between-source comparisons; complete factorial ANOVA results are in Supplementary Table S7a.

Coolaber preparations yielded 0.298 × 10^6^ to 0.785 × 10^6^ protoplasts/g fresh weight. Their maximum yield was obtained with the same 3.0% Cellulase R-10 and 2.0% Macerozyme R-10 combination but was approximately 5.9-fold lower than the maximum obtained with Yakult enzymes. Viability with Coolaber enzymes decreased from 96.90 ± 0.25% at the lowest enzyme combination to 68.90 ± 0.24% at 4.0% Cellulase R-10 and 2.0% Macerozyme R-10. Microscopy showed fewer intact protoplasts and more cellular debris in the Coolaber treatments (Figure 2).

The observed response to increasing one enzyme varied with the concentration of the other. At 1.0% Macerozyme R-10, increasing Cellulase R-10 from 2.0% to 4.0% reduced the Yakult yield from 3.529 × 10^6^ to 2.164 × 10^6^ protoplasts/g fresh weight. In contrast, at 4.0% Cellulase R-10, increasing Macerozyme R-10 from 1.0% to 2.0% increased yield to 4.251 × 10^6^ protoplasts/g fresh weight. The 3.0% Cellulase R-10 and 2.0% Macerozyme R-10 formulation was therefore selected for subsequent experiments.

### 3.2. Effect of Digestion Time on Protoplast Yield and Viability

**Figure 3:**
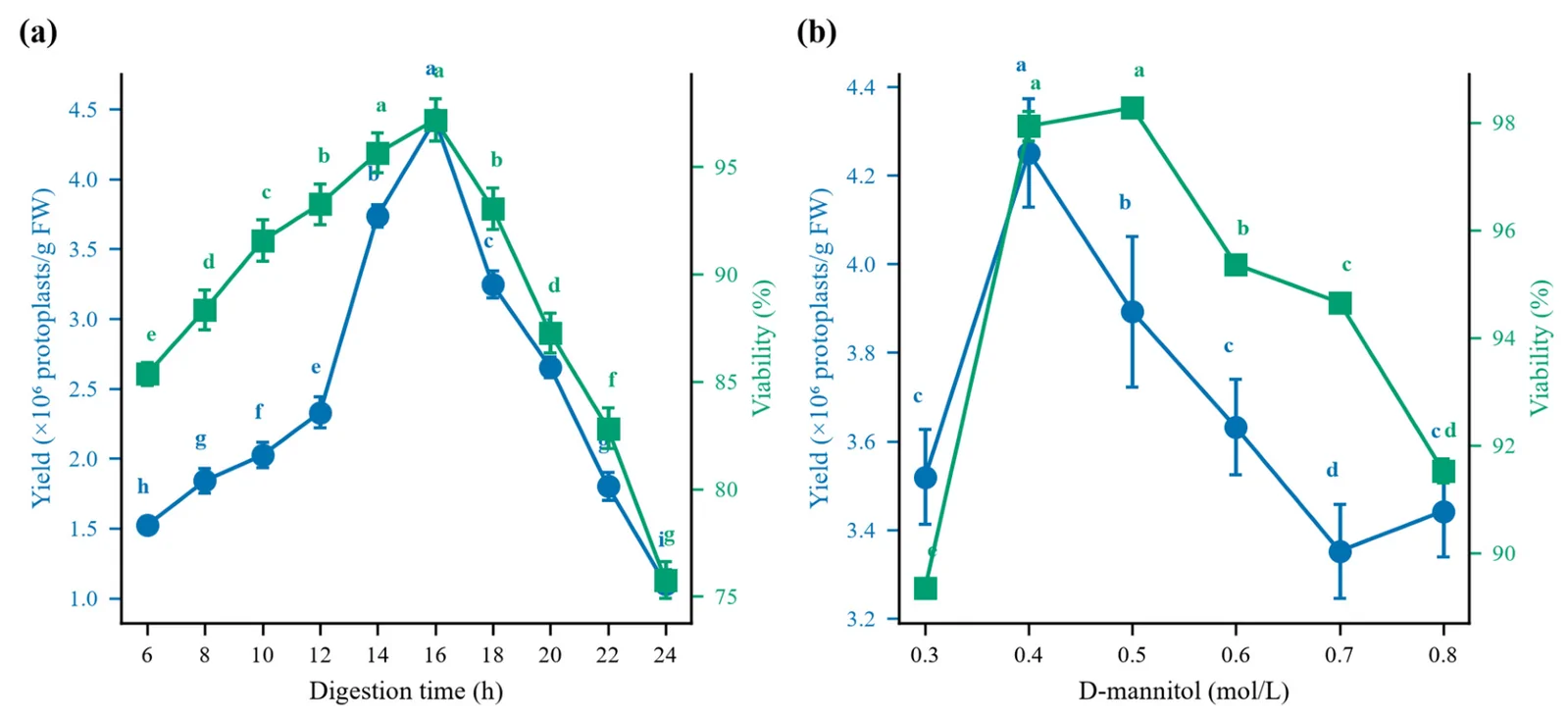
Protoplast recovery across digestion-time and D-mannitol treatments. (**a**) Yield and FDA viability after 6–24 h digestion. (**b**) Yield and viability at 0.3–0.8 mol/L D-mannitol. Data are mean ± SD from three plant-level biological replicates. Within each response and panel, means sharing a letter do not differ by Duncan’s multiple range test (*p* < 0.05).

Protoplast yield and viability varied significantly with digestion time (yield: *F*9,20 = 343.08, *p* < 0.001; viability: *F*9,20 = 154.47, *p* < 0.001; Figure 3a and Supplementary Table S7b). Yield increased from 1.522 ± 0.059 × 10^6^ protoplasts/g fresh weight at 6 h to 4.424 ± 0.152 × 10^6^ at 16 h, while viability increased from 85.35 ± 0.55% to 97.18 ± 0.99%. Both measures declined after 16 h. At 24 h, yield and viability were 1.100 ± 0.100 × 10^6^ protoplasts/g fresh weight and 75.76 ± 0.84%, respectively. A 16 h digestion was selected for the standard workflow. The lower yields at short digestion times may indicate incomplete protoplast release. Complete values and Duncan groupings are provided in Supplementary Table S2 and Figure 3a.

### 3.3. Effect of D-Mannitol Concentration on the Osmotic Response

Protoplast recovery differed significantly across D-mannitol concentrations (yield: *F*5,12 = 22.81, *p* < 0.001; viability: *F*5,12 = 955.17, *p* < 0.001; Figure 3b and Supplementary Table S7c). The highest yield, 4.251 ± 0.122 × 10^6^ protoplasts/g fresh weight, occurred at 0.4 mol/L, whereas the highest viability, 98.27 ± 0.17%, occurred at 0.5 mol/L and did not differ from 0.4 mol/L by Duncan’s test. At 0.3 mol/L, swelling, rupture, and debris were observed; at 0.7–0.8 mol/L, protoplasts showed shrinkage and irregular morphology. Because 0.4 mol/L maximized yield while maintaining high viability and regular morphology, it was selected. These responses are consistent with the need to maintain near-isotonic conditions [11]. Complete values and Duncan groupings are provided in Supplementary Table S2 and Figure 3b.

### 3.4. Effect of Cultivar on Protoplast Yield and Viability

**Figure 4:**
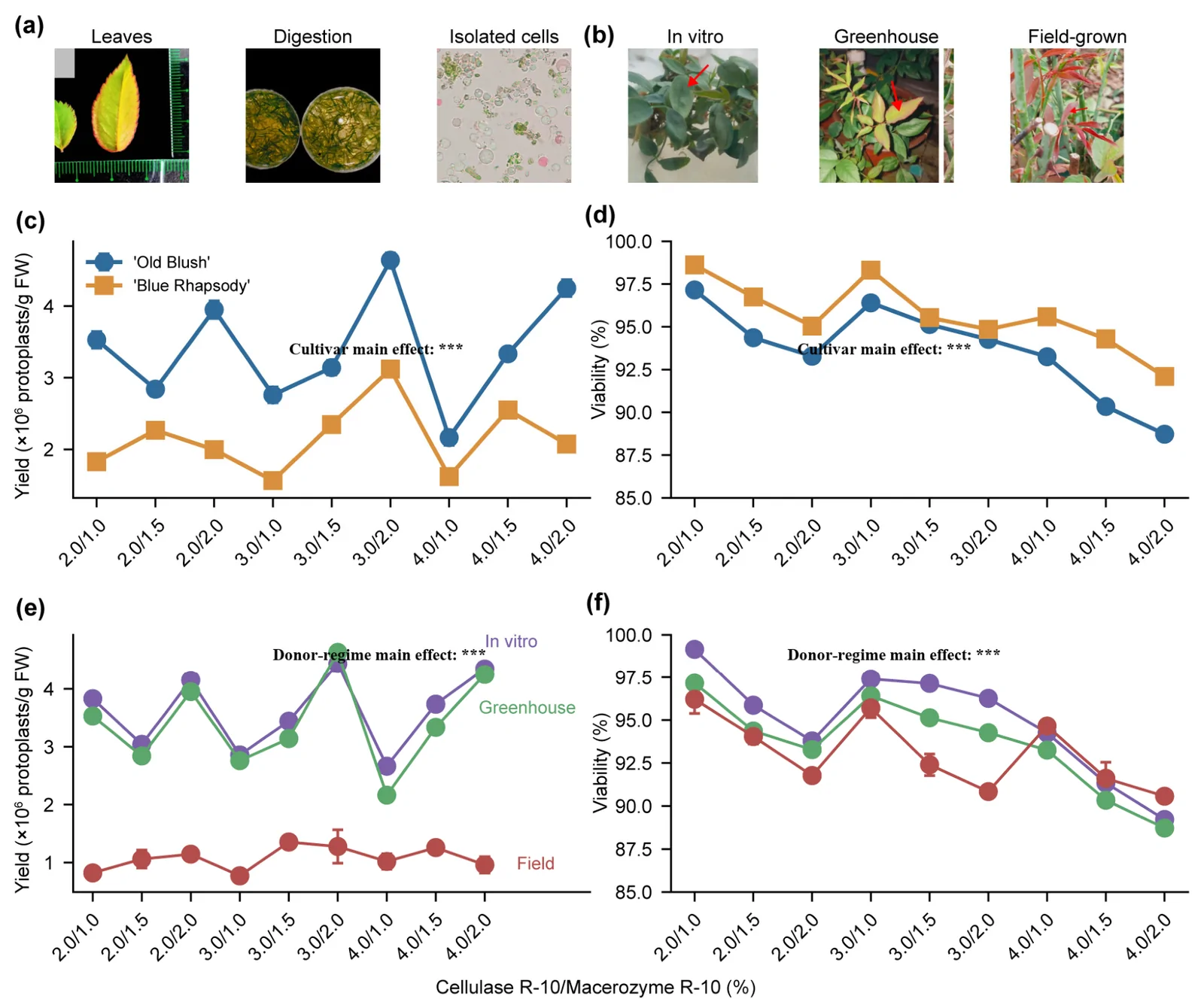
Protoplast isolation across cultivar and donor cultivation-regime comparisons. (**a**) Representative cultivar material and isolation images. (**b**) Representative donor-source records for in vitro plantlets, greenhouse cuttings, and field-grown plants; red arrows indicate the young, fully expanded leaves selected as donor tissue. (**c**,**d**) Yield and viability responses of ‘Old Blush’ and ‘Blue Rhapsody’. (**e**,**f**) Yield and viability responses of field-grown plants, in vitro plantlets, and greenhouse cuttings. Data are mean ± SD from three plant-level biological replicates. *** *p* < 0.001 for the indicated cultivar or donor-regime main effect in factorial ANOVA; complete results are in Supplementary Table S7d,e.

‘Old Blush’ and ‘Blue Rhapsody’ responded differently to the nine Yakult enzyme combinations (Figure 4c,d). Factorial ANOVA detected significant cultivar effects for yield (*F*1,36 = 2823.78, *p* < 0.001) and viability (*F*1,36 = 793.53, *p* < 0.001), as well as several cultivar × enzyme interactions (Supplementary Table S7d). ‘Old Blush’ yields ranged from 2.164 × 10^6^ to 4.641 × 10^6^ protoplasts/g fresh weight, whereas ‘Blue Rhapsody’ yields ranged from 1.564 × 10^6^ to 3.120 × 10^6^. Both cultivars produced their highest yield with 3.0% Cellulase R-10 and 2.0% Macerozyme R-10, but the ‘Blue Rhapsody’ maximum was 67.2% of the ‘Old Blush’ maximum. Complete values are provided in Supplementary Table S3.

‘Blue Rhapsody’ generally maintained higher viability, ranging from 92.09% to 98.61%, compared with 88.72% to 97.15% for ‘Old Blush’. From the lowest to the highest enzyme combination, viability decreased by 6.52 percentage points in ‘Blue Rhapsody’ and by 8.43 percentage points in ‘Old Blush’. Thus, the genotype providing the highest recovery was not the genotype maintaining the highest viability across enzyme treatments.

### 3.5. Effect of Donor Cultivation Regime on Protoplast Isolation

Protoplast yield and viability differed significantly among donor cultivation regimes (yield: *F*2,54 = 4826.07, *p* < 0.001; viability: *F*2,54 = 199.12, *p* < 0.001; Figure 4e,f and Supplementary Table S7e). Across the nine enzyme combinations, in vitro plantlets yielded 2.664 × 10^6^ to 4.441 × 10^6^ protoplasts/g fresh weight, and greenhouse cuttings yielded 2.164 × 10^6^ to 4.641 × 10^6^. At the selected formulation, their yields were similar, with greenhouse material producing the numerically highest condition-level value. Complete donor-regime values are provided in Supplementary Table S4.

Field-grown leaves yielded only 0.772 × 10^6^ to 1.355 × 10^6^ protoplasts/g fresh weight. Their maximum was 30.5% of the in vitro maximum and occurred at 3.0% Cellulase R-10 and 1.5% Macerozyme R-10 rather than at the formulation selected for controlled-environment material. Viability of recovered field protoplasts remained between 90.57% and 96.21%, indicating that the principal difference was recovery rather than complete loss of membrane integrity among the cells that were released. Greenhouse cuttings were selected for routine transfection because they combined high recovery with simpler propagation and repeated access to young leaves.

### 3.6. Full-Factorial Analysis Identified the Highest-Performing Tested PEG Condition

**Figure 5:**
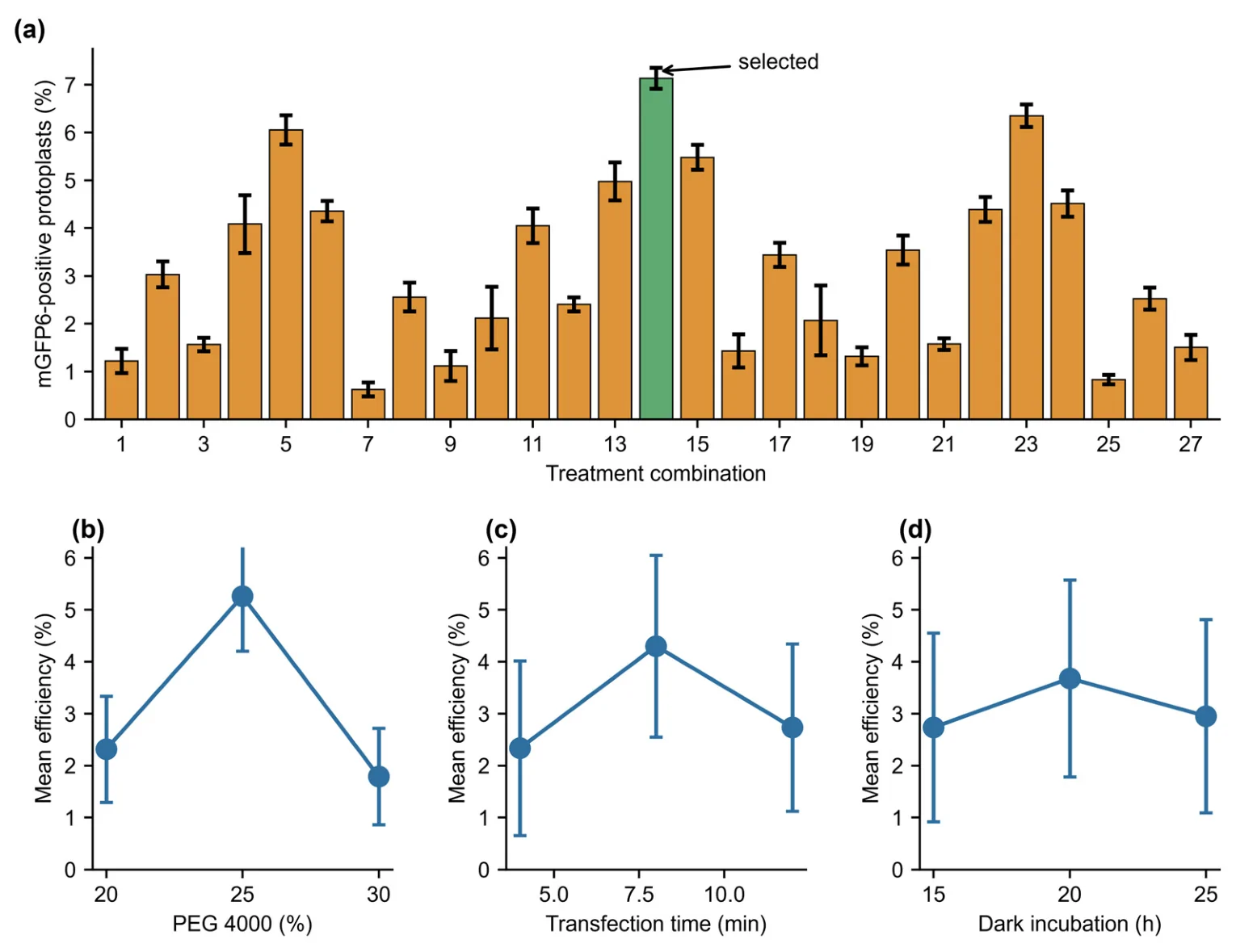
Factorial evaluation of PEG-mediated mGFP6 expression. (**a**) Transient expression efficiencies for all 27 combinations of PEG 4000 working-solution concentration, transfection time, and dark incubation time; bars show mean ± SD from three plant-level biological replicates. (**b**–**d**) Descriptive main-effect means ± SD across the nine treatment-level means at each factor level. The highest observed condition used 25% PEG 4000 working solution (12.5% nominal final concentration), 8 min transfection, and 20 h dark incubation.

Transient expression efficiency differed among the 27 combinations of PEG 4000 working-solution concentration, transfection time, and dark incubation time, ranging from 0.63 ± 0.15% to 7.14 ± 0.22% (Figure 5 and Supplementary Table S5). The highest observed efficiency within the tested design was obtained using 25% PEG 4000 working solution (12.5% nominal final PEG concentration in the reaction), 8 min transfection, and 20 h dark incubation. The lowest efficiency was observed using 30% PEG 4000 working solution, 4 min transfection, and 15 h incubation.

Across the other factors, mean efficiencies were 2.32%, 5.26%, and 1.79% for the 20%, 25%, and 30% PEG 4000 working-solution levels, respectively. Mean efficiencies were 2.34%, 4.30%, and 2.73% for 4, 8, and 12 min transfection, and 2.74%, 3.68%, and 2.95% for 15, 20, and 25 h incubation. Within the tested design, each factor showed an intermediate highest-performing level rather than a monotonic response.

Block-adjusted three-way ANOVA of arcsine-square-root-transformed transient expression efficiency showed significant main effects of PEG concentration, transfection time, and incubation time (all *p* < 0.001; Table 3), with F values of 993.20, 348.81, and 82.60, respectively. PEG concentration × transfection time was the only significant treatment-factor interaction (*F* = 7.18, *p* < 0.001); PEG concentration × incubation time, transfection time × incubation time, and the three-way interaction were not significant. The plant block was significant (*F* = 15.64, *p* < 0.001). The complete model table, including interpretation labels, is provided in Supplementary Table S6.

**Figure 6:**
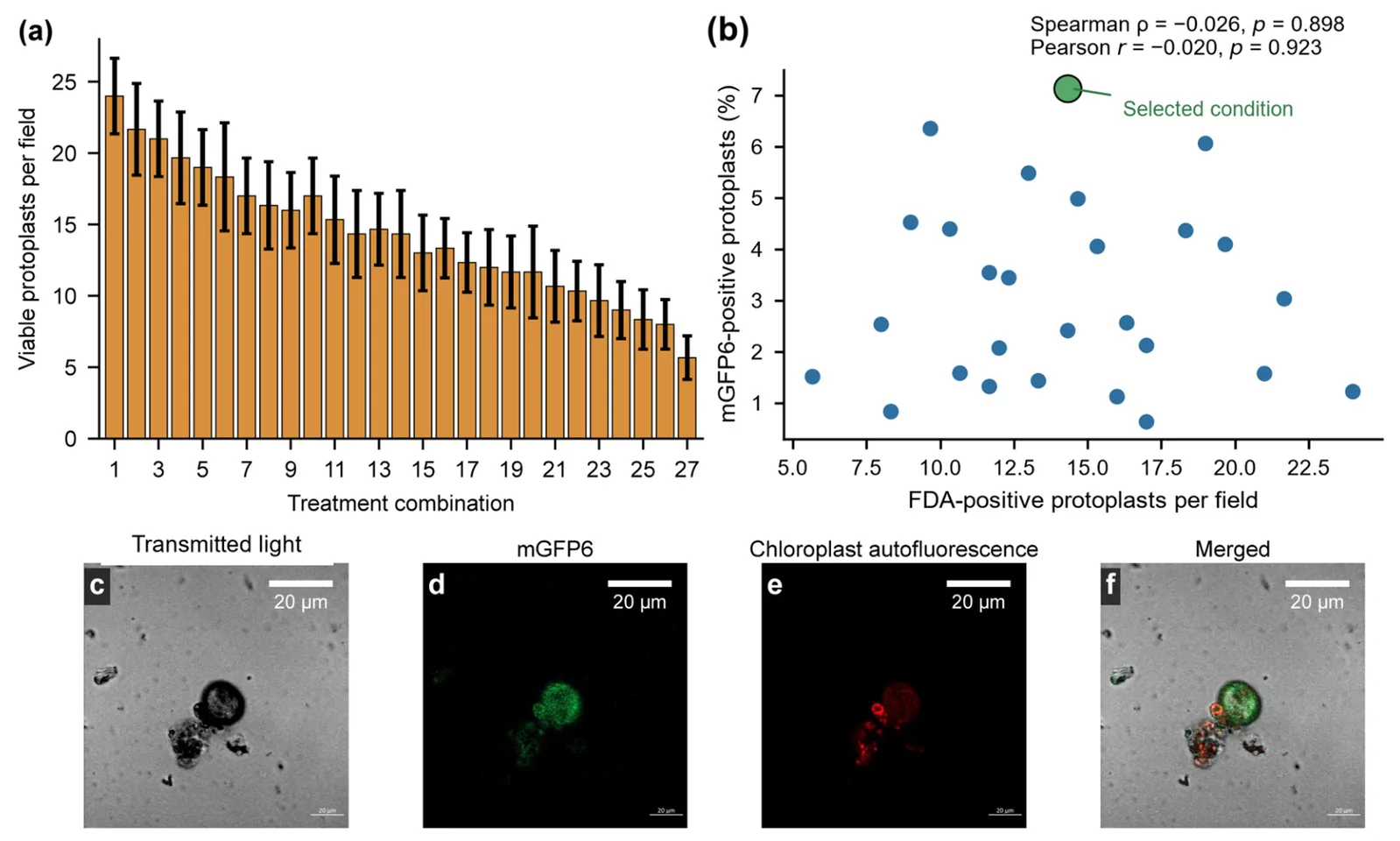
FDA-positive protoplast counts per field and reporter expression. (**a**) Post-transfection FDA-positive protoplast counts per imaged field; data are mean ± SD from three plant-level biological replicates. Counts are not survival percentages because a matched total-cell denominator was not recorded. (**b**) Descriptive relationship between FDA-positive count per field and mGFP6-positive fraction across the 27 treatment-level means; the selected condition is shown in green. (**c**–**f**) Representative transmitted-light, mGFP6, chloroplast-autofluorescence, and merged confocal channels. Scale bars, 20 μm.

Confocal microscopy detected mGFP6 fluorescence in treated protoplasts under the selected condition (Figure 6 and Figure S1a–e). The GFP channel, chloroplast autofluorescence channel, transmitted-light image, and merged image separated reporter fluorescence from chloroplast signal in representative fields. The available matched empty-vector control images showed GFP-channel background and chloroplast/autofluorescence signal, but no distinct mGFP6-positive protoplast pattern (Supplementary Figure S1f–h).

### 3.7. FDA-Positive Protoplast Counts per Field and Reporter Expression

FDA-positive protoplast counts ranged from 5.67 ± 1.53 to 24.00 ± 2.65 cells per imaged field (Figure 6 and Supplementary Table S5). This count is influenced by suspension density and field selection and is not a survival percentage. The treatment using 20% PEG 4000 working solution for 4 min followed by 15 h incubation produced 24.00 ± 2.65 FDA-positive protoplasts per field but only 1.22 ± 0.25% mGFP6-positive protoplasts. The selected expression condition produced 14.33 ± 3.06 FDA-positive protoplasts per field together with the highest mGFP6-positive fraction.

Across the 27 treatment-level means, FDA-positive protoplast count per field and mGFP6-positive fraction yielded near-zero descriptive correlations (Spearman ρ = −0.026, *p* = 0.898; Pearson *r* = −0.020, *p* = 0.923; Figure 6b). Because the FDA endpoint lacked a total-cell denominator and was sensitive to field density, this analysis does not quantify survival or establish a survival–expression trade-off.

## 4. Discussion

We evaluated a mesophyll protoplast isolation and transient expression workflow for *R. chinensis* ‘Old Blush’. Previous studies established the feasibility of isolating, culturing, and regenerating rose protoplasts from mesophyll or embryogenic tissues [7,8,9]. By examining donor cultivation regime, genotype, specific enzyme preparations and formulations, digestion time, osmotic conditions, and PEG-mediated transfection within one framework, this study identifies selected operating conditions for the tested material rather than a universal rose protocol.

The specific enzyme preparations tested were associated with substantial variation: the maximum yield with Yakult preparations was about 5.9-fold greater than that with the corresponding Coolaber treatment. Comparable woody-plant protocols also report strong dependence on enzyme composition and tissue context [17,18,19]. The biochemical cause of the present source effect was not measured. Differences in activity, formulation, storage, batch properties, accessory activities, or residual impurities are possible, but direct assays would be required to distinguish them. Source, product, and batch should therefore be reported and activity rechecked when either changes.

The concentration matrix showed that Cellulase R-10 and Macerozyme R-10 could not be interpreted independently, consistent with the significant interactions in Supplementary Table S7a. Increasing cellulase did not improve recovery when macerozyme remained low, whereas increasing macerozyme at high cellulase increased yield. This pattern is compatible with complementary degradation of cellulose-rich walls and the pectin-rich middle lamella, although wall composition and enzyme activity were not measured. Similar empirical enzyme-matrix optimization has been required in *Populus*, *Liriodendron*, and *Catalpa* [17,18,19].

Digestion time and osmotic conditions defined a narrow operating range. The lower yields at short times may reflect incomplete release, whereas prolonged digestion decreased both yield and viability. D-mannitol at 0.4–0.5 mol/L maintained high viability and regular morphology; lower and higher concentrations were associated with rupture or shrinkage. These observations agree with the need to maintain osmotic balance [11] and with species-specific optimization in woody mesophyll systems [17,18,19].

Recovery varied significantly among donor cultivation regimes. Controlled-environment plantlets and greenhouse cuttings yielded more protoplasts than field-grown leaves, although released field protoplasts remained highly viable. Donor age and physiological state are recognized determinants of woody-plant protoplast performance [17,18,19], but cuticle thickness, wall composition, and lignification were not measured here and remain possible explanations. Greenhouse cuttings combined high recovery with repeated access to young leaves without sterile-culture maintenance.

‘Old Blush’ and ‘Blue Rhapsody’ also differed significantly: ‘Old Blush’ produced higher yields, whereas ‘Blue Rhapsody’ maintained higher marginal viability. Earlier Rosa work similarly demonstrated genotype dependence in protoplast isolation and regeneration [7,8,9]. Cell-wall or membrane properties may contribute, but no biochemical measurements were made. The selected conditions should therefore be re-evaluated for every additional cultivar.

The full-factorial experiment placed intermediate PEG working-solution concentration and exposure time above the lower and higher tested levels. Similar non-linear responses have been reported in herbaceous and woody protoplast workflows [4,12,13,14,15,16,17,18,19]. In ‘Old Blush’, 25% PEG 4000 working solution (12.5% nominal final concentration) for 8 min followed by 20 h dark incubation produced 7.14 ± 0.22% mGFP6-positive protoplasts. This efficiency is adequate for detecting and imaging individually positive cells in the present experiment, but it is substantially below efficiencies commonly sought for population-level biochemical assays or high-throughput screening [17]. It should not be extrapolated to promoter libraries, genome-editing evaluation, or bulk molecular assays without further optimization and validation.

Across the 27 treatment-level means, FDA-positive protoplast counts per imaged field were not associated with mGFP6-positive fractions by Pearson or Spearman analysis. The selected condition is therefore the highest-expression condition observed in the tested design, not evidence of a quantified survival-expression trade-off or a membrane-permeabilization mechanism. Establishing a mechanism would require direct permeability probes, labeled DNA uptake, oxidative-damage measurements, or time-resolved imaging.

The study has several limitations. PEG-mediated transfection was evaluated in one principal genotype, with one stand-alone mGFP6 reporter and material collected on one experimental date; independent-date reproducibility was not tested. Isolation variables were evaluated sequentially, and enzyme performance may vary with source, formulation, and batch. The post-transfection FDA endpoint was a density- and field-dependent count rather than a survival percentage, and its association with expression was descriptive. Protoplast division, regeneration, stable transformation, cross-laboratory reproducibility, population-level biochemical assays, high-throughput promoter assays, and genome-editing evaluation were not demonstrated. Transfer to other genotypes, reporters, dates, laboratories, or downstream applications therefore requires empirical validation.

## 5. Conclusions

For *Rosa chinensis* ‘Old Blush’, greenhouse-grown young leaves digested for 16 h with 3.0% Cellulase R-10, 2.0% Macerozyme R-10, and 0.4 mol/L D-mannitol provided the selected isolation conditions. Within the 27-treatment factorial grid, 25% PEG 4000 working solution (12.5% nominal final concentration) for 8 min followed by 20 h dark incubation produced the highest observed expression (7.14 ± 0.22% mGFP6-positive protoplasts). This study provides a useful starting protocol for PEG-mediated transient expression and imaging of individually positive protoplasts in ‘Old Blush’ under the tested conditions. It does not establish transferability, independent-date reproducibility, population-level applications, protoplast division, plant regeneration, or stable transformation.

## Figures and Tables

**Table 1:** Experimental factors and levels.

Experiment	Factor	Levels
Enzyme formulation	Cellulase R-10	2.0%, 3.0%, 4.0% (*w*/*v*)
Enzyme formulation	Macerozyme R-10	1.0%, 1.5%, 2.0% (*w*/*v*)
Enzyme preparation source	Source	Yakult; Coolaber
Digestion time	Time	6, 8, 10, 12, 14, 16, 18, 20, 22, 24 h
Osmotic condition	D-mannitol	0.3, 0.4, 0.5, 0.6, 0.7, 0.8 mol/L
Genotype	Cultivar	‘Old Blush’; ‘Blue Rhapsody’
Donor source	Cultivation regime	In vitro; greenhouse cutting; field-grown
Transfection	PEG 4000 working solution	20%, 25%, 30% (*w*/*v*; nominal final reaction concentrations 10%, 12.5%, 15%)
Transfection	Exposure time	4, 8, 12 min
Transfection	Dark incubation	15, 20, 25 h

**Table 2:** Selected operating conditions and outcomes.

Component	Selected Condition	Outcome
Enzyme formulation	3.0% Cellulase R-10 + 2.0% Macerozyme R-10 (Yakult)	4.641 ± 0.102 × 10^6^ protoplasts/g FW; 94.26 ± 0.15% viability
Digestion time	16 h	4.424 ± 0.152 × 10^6^ protoplasts/g FW; 97.18 ± 0.99% viability
Osmoticum	0.4 mol/L D-mannitol	4.251 ± 0.122 × 10^6^ protoplasts/g FW; 97.93 ± 0.27% viability
PEG-mediated transfection	25% PEG 4000 working solution (12.5% nominal final concentration), 8 min, 20 h dark incubation	7.14 ± 0.22% mGFP6-positive protoplasts

**Table 3:** Block-adjusted three-way ANOVA of arcsine-square-root-transformed transient expression efficiency.

Source	Sum of Squares	df	Mean Square	F	*p*
PEG 4000 working-solution concentration	0.154009	2	0.077004	993.20	<0.001
Transfection time	0.054087	2	0.027043	348.81	<0.001
Incubation time	0.012809	2	0.006404	82.60	<0.001
PEG concentration × transfection time	0.002228	4	0.000557	7.18	<0.001
PEG concentration × incubation time	0.000345	4	0.000086	1.11	0.361
Transfection time × incubation time	0.000153	4	0.000038	0.49	0.741
PEG concentration × transfection time × incubation time	0.000559	8	0.000070	0.90	0.523
Plant block	0.002425	2	0.001213	15.64	<0.001
Residual	0.004032	52	0.000078		

## Data Availability

The data presented in this study are available in the article and Supplementary Materials. The sequence-derived pMDC85-1–mGFP6 map and complete annotated plasmid sequence are provided as Supplementary Figure S2 and Supplementary Data S1, respectively. No new organismal gene sequence was generated in this study. Additional non-public raw data supporting the findings are available from the corresponding author upon reasonable request.

## Supplementary Materials

The following supporting information can be downloaded at: https://www.mdpi.com/article/10.3390/plants15182739/s1, Table S1: Effects of enzyme preparation source and concentration on ‘Old Blush’ protoplast yield and viability; Table S2: Effects of digestion time and D-mannitol concentration; Table S3: Comparison of ‘Old Blush’ and ‘Blue Rhapsody’; Table S4: Effects of donor cultivation regime; Table S5: Full-factorial PEG-mediated transfection dataset; Table S6: Block-adjusted three-way ANOVA of arcsine-square-root-transformed transient expression efficiency; Table S7: Inferential analyses of protoplast isolation experiments; Figure S1: Representative mGFP6 fluorescence and matched empty-vector control. (a–e) Representative mGFP6-positive protoplast fields after PEG-mediated transient expression. (f–h) Matched pMDC85-1 empty-vector-transfected control images showing GFP-channel background, chloroplast autofluorescence, and merged control signal. Scale bars: (a–e), 20 μm; (f–h), 100 μm; Figure S2: Sequence-derived map of the 12,462-bp pMDC85-1–mGFP6 reporter construct. The map was generated from the author-supplied SnapGene file. The construct contains the CaMV 35S–Gateway–mGFP6–6×His expression region, T-DNA borders, HygR, KanR, the chloramphenicol-resistance/ccdB Gateway cassette, replication functions, and bacterial origin of replication; Data S1: Complete annotated sequence of pMDC85-1–mGFP6 in GenBank format (Supplementary_Data_S1_pMDC85-1_mGFP6.gb). No new organismal gene sequence was generated in this study.
