# Supplementary material for: Systematic Evaluation of Mesophyll Protoplast Isolation and PEG-Mediated Transient Expression in Rosa chinensis ‘Old Blush’
Source: Plants (Basel). 2026 Sep 8;15(18):2739. doi: 10.3390/plants15182739 (PMC13610953; doi:10.3390/plants15182739)
Supplement: Supplementary file 1 [file plants-15-02739-s001.zip › Zhao_et_al_Plants_Supplementary_Information_Revised_Clean.pdf]

Supplementary Materials

Systematic evaluation of mesophyll protoplast isolation and PEG-mediated transient expression in *Rosa chinensis* 'Old Blush'

Supplementary Table S1. Effects of enzyme preparation source and concentration on 'Old Blush' protoplast yield and viability

Preparation source	Cellulase R-10 (%)	Macerozyme R-10 (%)	Yield (× 10^6 protoplasts/g FW)	Viability (%)
Yakult	2.0	1.0	3.529 ± 0.116	97.15 ± 0.17
Yakult	2.0	1.5	2.840 ± 0.096	94.36 ± 0.15
Yakult	2.0	2.0	3.951 ± 0.122	93.28 ± 0.14
Yakult	3.0	1.0	2.759 ± 0.110	96.40 ± 0.18
Yakult	3.0	1.5	3.141 ± 0.102	95.13 ± 0.13
Yakult	3.0	2.0	4.641 ± 0.102	94.26 ± 0.15
Yakult	4.0	1.0	2.164 ± 0.106	93.25 ± 0.17
Yakult	4.0	1.5	3.334 ± 0.082	90.34 ± 0.16
Yakult	4.0	2.0	4.251 ± 0.117	88.72 ± 0.18
Coolaber	2.0	1.0	0.521 ± 0.011	96.90 ± 0.25
Coolaber	2.0	1.5	0.385 ± 0.009	92.50 ± 0.22
Coolaber	2.0	2.0	0.612 ± 0.012	88.80 ± 0.20
Coolaber	3.0	1.0	0.368 ± 0.011	94.80 ± 0.24
Coolaber	3.0	1.5	0.436 ± 0.010	85.60 ± 0.19
Coolaber	3.0	2.0	0.785 ± 0.010	81.30 ± 0.21
Coolaber	4.0	1.0	0.298 ± 0.010	90.10 ± 0.23
Coolaber	4.0	1.5	0.479 ± 0.008	77.50 ± 0.20
Coolaber	4.0	2.0	0.716 ± 0.011	68.90 ± 0.24

Data are mean ± SD from three plant-level biological replicates. Inferential results are provided in Supplementary Table S7.

Supplementary Table S2. Effects of digestion time and D-mannitol concentration.

Within each response and subtable, means sharing a lowercase letter do not differ by Duncan's multiple range test ($P < 0.05$).

S2a. Digestion time

Digestion time (h)	Yield (× 10^6 protoplasts/g FW)	Viability (%)

Digestion time (h)	Yield (× 10^6 protoplasts/g FW)	Viability (%)
6	1.522 ± 0.059 h	85.35 ± 0.55 e
8	1.839 ± 0.088 g	88.34 ± 0.93 d
10	2.023 ± 0.092 f	91.55 ± 0.97 c
12	2.329 ± 0.110 e	93.26 ± 0.95 b
14	3.737 ± 0.082 b	95.64 ± 0.93 a
16	4.424 ± 0.152 a	97.18 ± 0.99 a
18	3.244 ± 0.096 c	93.05 ± 0.97 b
20	2.651 ± 0.073 d	87.25 ± 0.92 d
22	1.800 ± 0.100 g	82.82 ± 0.95 f
24	1.100 ± 0.100 i	75.76 ± 0.84 g

S2b. D-mannitol concentration

D-mannitol (mol/L)	Yield (× 10^6 protoplasts/g FW)	Viability (%)
0.3	3.519 ± 0.107 c	89.34 ± 0.18 e
0.4	4.251 ± 0.122 a	97.93 ± 0.27 a
0.5	3.892 ± 0.170 b	98.27 ± 0.17 a
0.6	3.631 ± 0.108 c	95.36 ± 0.14 b
0.7	3.352 ± 0.106 d	94.65 ± 0.18 c
0.8	3.442 ± 0.104 c	91.52 ± 0.22 d

Supplementary Table S3. Comparison of 'Old Blush' and 'Blue Rhapsody'

Cellulase (%)	Macerozyme (%)	'Blue Rhapsody' yield (× 10^6 protoplasts/g FW)	'Old Blush' yield (× 10^6 protoplasts/g FW)	'Blue Rhapsody' viability (%)	'Old Blush' viability (%)
2.0	1.0	1.828 ± 0.023	3.529 ± 0.116	98.61 ± 0.14	97.15 ± 0.17
2.0	1.5	2.267 ± 0.047	2.840 ± 0.096	96.74 ± 0.36	94.36 ± 0.15
2.0	2.0	1.996 ± 0.029	3.951 ± 0.122	95.04 ± 0.43	93.28 ± 0.14
3.0	1.0	1.564 ± 0.078	2.759 ± 0.110	98.31 ± 0.31	96.40 ± 0.18
3.0	1.5	2.346 ± 0.067	3.141 ± 0.102	95.53 ± 0.35	95.13 ± 0.13
3.0	2.0	3.120 ± 0.029	4.641 ± 0.102	94.85 ± 0.33	94.26 ± 0.15
4.0	1.0	1.619 ± 0.067	2.164 ± 0.106	95.58 ± 0.33	93.25 ± 0.17

Cellulase (%)	Macerozyme (%)	'Blue Rhapsody' yield (× 10^6 protoplasts/g FW)	'Old Blush' yield (× 10^6 protoplasts/g FW)	'Blue Rhapsody' viability (%)	'Old Blush' viability (%)
4.0	1.5	2.551 ± 0.067	3.334 ± 0.082	94.29 ± 0.34	90.34 ± 0.16
4.0	2.0	2.073 ± 0.091	4.251 ± 0.117	92.09 ± 0.36	88.72 ± 0.18

Yields are × 10^6 protoplasts/g FW. Data are mean ± SD from three plant-level biological replicates.

Supplementary Table S4. Effects of donor cultivation regime

Cellulase (%)	Macerozyme (%)	In vitro yield (× 10^6 protoplasts/g FW)	Greenhouse yield (× 10^6 protoplasts/g FW)	Field yield (× 10^6 protoplasts/g FW)	In vitro viability (%)	Greenhouse viability (%)	Field viability (%)
2.0	1.0	3.829 ± 0.028	3.529 ± 0.116	0.822 ± 0.090	99.15 ± 0.17	97.15 ± 0.17	96.21 ± 0.83
2.0	1.5	3.040 ± 0.056	2.840 ± 0.096	1.061 ± 0.156	95.86 ± 0.15	94.36 ± 0.15	94.04 ± 0.43
2.0	2.0	4.151 ± 0.018	3.951 ± 0.122	1.146 ± 0.085	93.78 ± 0.14	93.28 ± 0.14	91.77 ± 0.37
3.0	1.0	2.859 ± 0.112	2.759 ± 0.110	0.772 ± 0.078	97.40 ± 0.18	96.40 ± 0.18	95.70 ± 0.56
3.0	1.5	3.441 ± 0.054	3.141 ± 0.102	1.355 ± 0.047	97.13 ± 0.13	95.13 ± 0.13	92.40 ± 0.63
3.0	2.0	4.441 ± 0.026	4.641 ± 0.102	1.278 ± 0.287	96.26 ± 0.15	94.26 ± 0.15	90.84 ± 0.35
4.0	1.0	2.664 ± 0.048	2.164 ± 0.106	1.020 ± 0.134	94.25 ± 0.17	93.25 ± 0.17	94.65 ± 0.26
4.0	1.5	3.734 ± 0.057	3.334 ± 0.082	1.259 ± 0.052	91.34 ± 0.16	90.34 ± 0.16	91.61 ± 0.93
4.0	2.0	4.351 ± 0.023	4.251 ± 0.117	0.961 ± 0.141	89.22 ± 0.18	88.72 ± 0.18	90.57 ± 0.18

Yields are × 10^6 protoplasts/g FW. Data are mean ± SD from three plant-level biological replicates.

Supplementary Table S5. Full-factorial PEG-mediated transfection dataset

Treatment	PEG 4000 working solution (%)	Transfection (min)	Dark incubation (h)	Transient expression efficiency (%)	FDA-positive protoplasts per imaged field
1	20	4	15	1.22 ± 0.25	24.00 ± 2.65
2	20	8	15	3.03 ± 0.27	21.67 ± 3.21
3	20	12	15	1.57 ± 0.14	21.00 ± 2.65
4	25	4	15	4.09 ± 0.61	19.67 ± 3.21
5	25	8	15	6.06 ± 0.31	19.00 ± 2.65
6	25	12	15	4.36 ± 0.22	18.33 ± 3.79
7	30	4	15	0.63 ± 0.15	17.00 ± 2.65
8	30	8	15	2.56 ± 0.30	16.33 ± 3.06

Treatment	PEG 4000 working solution (%)	Transfection (min)	Dark incubation (h)	Transient expression efficiency (%)	FDA-positive protoplasts per imaged field
9	30	12	15	1.12 ± 0.31	16.00 ± 2.65
10	20	4	20	2.12 ± 0.65	17.00 ± 2.65
11	20	8	20	4.05 ± 0.36	15.33 ± 3.06
12	20	12	20	2.41 ± 0.15	14.33 ± 3.06
13	25	4	20	4.98 ± 0.40	14.67 ± 2.52
14	25	8	20	7.14 ± 0.22	14.33 ± 3.06
15	25	12	20	5.48 ± 0.26	13.00 ± 2.65
16	30	4	20	1.43 ± 0.35	13.33 ± 2.08
17	30	8	20	3.44 ± 0.25	12.33 ± 2.08
18	30	12	20	2.07 ± 0.73	12.00 ± 2.65
19	20	4	25	1.32 ± 0.19	11.67 ± 2.52
20	20	8	25	3.54 ± 0.30	11.67 ± 3.21
21	20	12	25	1.58 ± 0.12	10.67 ± 2.52
22	25	4	25	4.39 ± 0.26	10.33 ± 2.08
23	25	8	25	6.35 ± 0.24	9.67 ± 2.52
24	25	12	25	4.52 ± 0.27	9.00 ± 2.00
25	30	4	25	0.83 ± 0.10	8.33 ± 2.08
26	30	8	25	2.53 ± 0.23	8.00 ± 1.73
27	30	12	25	1.51 ± 0.26	5.67 ± 1.53

PEG values are working-solution concentrations. The post-transfection endpoint is reported as FDA-positive protoplasts per imaged field. Data are mean ± SD from three plant-level biological replicates.

Supplementary Table S6. Block-adjusted three-way ANOVA of arcsine-square-root-transformed transient expression efficiency

Source	Sum of squares	df	Mean square	F	P	Interpretation
PEG 4000 working-solution concentration	0.154009	2	0.077004	993.20	<0.001	Significant
Transfection time	0.054087	2	0.027043	348.81	<0.001	Significant
Incubation time	0.012809	2	0.006404	82.60	<0.001	Significant
PEG concentration × transfection time	0.002228	4	0.000557	7.18	<0.001	Significant
PEG concentration × incubation time	0.000345	4	0.000086	1.11	0.361	Not significant
Transfection time × incubation time	0.000153	4	0.000038	0.49	0.741	Not significant

Source	Sum of squares	df	Mean square	F	P	Interpretation
PEG concentration × transfection time × incubation time	0.000559	8	0.000070	0.90	0.523	Not significant
Plant block	0.002425	2	0.001213	15.64	<0.001	Significant
Residual	0.004032	52	0.000078			

Supplementary Table S7. Inferential analyses of protoplast isolation experiments

Factorial ANOVA included all main effects and interactions. Digestion time and D-mannitol concentration were analyzed by one-way ANOVA. Plant was the biological unit (n = 3 per condition). df is reported as numerator, denominator; P values below 0.001 are shown as <0.001.

S7a. Enzyme preparation source × Cellulase R-10 × Macerozyme R-10

Outcome	Source	df	F	P
Yield	Preparation source	1, 36	19692.04	<0.001
Yield	Cellulase	2, 36	17.89	<0.001
Yield	Macerozyme	2, 36	699.57	<0.001
Yield	Preparation source × Cellulase	2, 36	11.72	<0.001
Yield	Preparation source × Macerozyme	2, 36	290.20	<0.001
Yield	Cellulase × Macerozyme	4, 36	110.25	<0.001
Yield	Preparation source × Cellulase × Macerozyme	4, 36	51.21	<0.001
Viability	Preparation source	1, 36	19827.72	<0.001
Viability	Cellulase	2, 36	10476.60	<0.001
Viability	Macerozyme	2, 36	9759.83	<0.001
Viability	Preparation source × Cellulase	2, 36	2909.44	<0.001
Viability	Preparation source × Macerozyme	2, 36	3543.76	<0.001
Viability	Cellulase × Macerozyme	4, 36	521.65	<0.001
Viability	Preparation source × Cellulase × Macerozyme	4, 36	419.77	<0.001

S7b. Digestion time

Outcome	Source	df	F	P
Yield	Digestion time	9, 20	343.08	<0.001
Viability	Digestion time	9, 20	154.47	<0.001

S7c. D-mannitol concentration

Outcome	Source	df	F	P

Outcome	Source	df	F	P
Yield	D-mannitol concentration	5, 12	22.81	<0.001
Viability	D-mannitol concentration	5, 12	955.17	<0.001

S7d. Cultivar × Cellulase R-10 × Macerozyme R-10

Outcome	Source	df	F	P
Yield	Cultivar	1, 36	2823.78	<0.001
Yield	Cellulase	2, 36	44.82	<0.001
Yield	Macerozyme	2, 36	724.20	<0.001
Yield	Cultivar × Cellulase	2, 36	11.59	<0.001
Yield	Cultivar × Macerozyme	2, 36	210.24	<0.001
Yield	Cellulase × Macerozyme	4, 36	149.13	<0.001
Yield	Cultivar × Cellulase × Macerozyme	4, 36	40.73	<0.001
Viability	Cultivar	1, 36	793.53	<0.001
Viability	Cellulase	2, 36	1019.15	<0.001
Viability	Macerozyme	2, 36	814.94	<0.001
Viability	Cultivar × Cellulase	2, 36	83.41	<0.001
Viability	Cultivar × Macerozyme	2, 36	2.51	0.096
Viability	Cellulase × Macerozyme	4, 36	10.80	<0.001
Viability	Cultivar × Cellulase × Macerozyme	4, 36	15.77	<0.001

S7e. Donor regime × Cellulase R-10 × Macerozyme R-10

Outcome	Source	df	F	P
Yield	Donor regime	2, 54	4826.07	<0.001
Yield	Cellulase	2, 54	7.01	0.002
Yield	Macerozyme	2, 54	601.00	<0.001
Yield	Donor regime × Cellulase	4, 54	6.76	<0.001
Yield	Donor regime × Macerozyme	4, 54	115.47	<0.001
Yield	Cellulase × Macerozyme	4, 54	101.48	<0.001
Yield	Donor regime × Cellulase × Macerozyme	8, 54	28.51	<0.001
Viability	Donor regime	2, 54	199.12	<0.001
Viability	Cellulase	2, 54	917.26	<0.001

Outcome	Source	df	F	P
Viability	Macerozyme	2, 54	882.07	<0.001
Viability	Donor regime × Cellulase	4, 54	115.67	<0.001
Viability	Donor regime × Macerozyme	4, 54	5.33	0.001
Viability	Cellulase × Macerozyme	4, 54	22.09	<0.001
Viability	Donor regime × Cellulase × Macerozyme	8, 54	14.00	<0.001

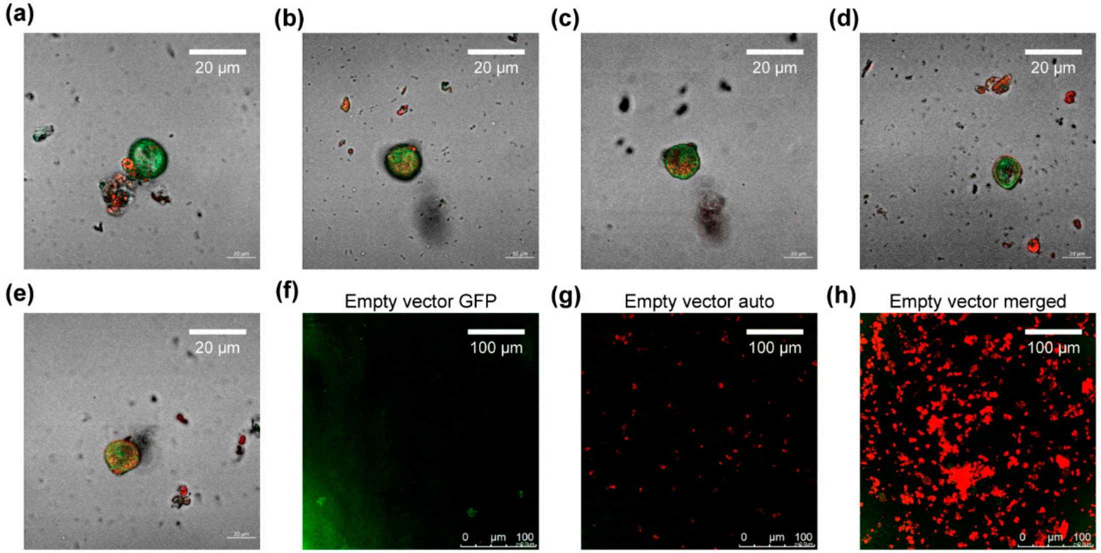

Supplementary Figure S1. Representative mGFP6 fluorescence and matched empty-vector control. (a-e) Representative mGFP6-positive protoplast fields after PEG-mediated transient expression. (f-h) Matched pMDC85-1 empty-vector-transfected control images showing GFP-channel background, chloroplast autofluorescence, and merged control signal. Scale bars: a-e, 20 µm; f-h, 100 µm.

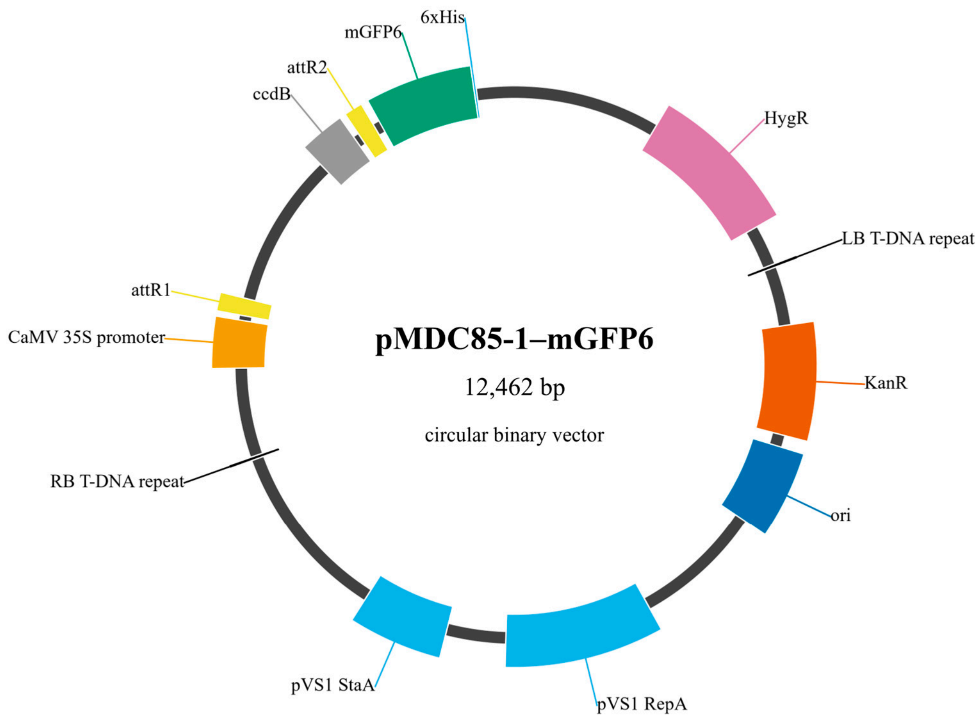

Map generated from the author-supplied SnapGene sequence; feature positions are sequence-derived.

Supplementary Figure S2. Sequence-derived map of the 12,462-bp pMDC85-1–mGFP6 reporter construct. The map was generated from the author-supplied SnapGene file. The construct contains the CaMV 35S–Gateway–mGFP6–6×His expression region, T-DNA borders, HygR, KanR, the chloramphenicol-resistance/ccdB Gateway cassette, replication functions, and bacterial origin of replication. The complete annotated sequence is provided as Supplementary Data S1.

Supplementary Data S1. Complete annotated sequence of pMDC85-1–mGFP6 in GenBank format (Supplementary_Data_S1_pMDC85-1_mGFP6.gb). No new organismal gene sequence was generated in this study.
